# Quality Improvement Initiative to Reduce Inappropriate Viral Testing within the Pediatric Emergency Department

**DOI:** 10.1097/pq9.0000000000000898

**Published:** 2026-07-16

**Authors:** Cherisse Mecham, Rachel Kortman, Emma Albrecht, Sam Dowell, Cecilia Monteilh

**Affiliations:** From the * Division of Emergency Medicine, Phoenix Children’s, Phoenix, Ariz.; † Graduate Medical Education, Phoenix Children’s, Phoenix, Ariz.

## Abstract

**Introduction:**

The American Academy of Pediatrics Section on Emergency Medicine’s Choosing Wisely Campaign includes recommendations discouraging comprehensive viral panel testing in children with suspected respiratory viral illness. In the year preceding project implementation, our free-standing pediatric emergency department (PED) ordered comprehensive respiratory pathogen panels (RPPs) in 11.9% of all visits, and 50% of those lacked a clinical indication. This project aimed to reduce the percentage of PED visits with RPP orders from 11.9% to 9% over a 6-month viral respiratory season.

**Methods:**

We conducted four major interventions over 2 plan-do-study-act cycles. Interventions included developing a clinical guideline for viral testing, performing provider education, introducing a narrower-spectrum Quad (influenza A/B, COVID-19, and respiratory syncytial virus) polymerase chain reaction (PCR) test, and adding an order-entry indication requirement for RPPs, which also served as a just-in-time educational tool for providers. We tracked data via a clinical dashboard and through periodic chart reviews.

**Results:**

Of the 43,283 patient visits during the project period, the percentage of PED RPP orders decreased from 11.9% at baseline to 6.8% during the intervention period and remained at 6.8% in the following respiratory season. The project had sustained cost savings of over $300,000 in RPP testing each year. The decrease in RPP orders did not lead to an increase in the cumulative use of other viral tests.

**Conclusions:**

This quality improvement initiative significantly improved and sustained comprehensive viral PCR testing practices in the PED without increasing the use of alternative respiratory viral testing.

## INTRODUCTION

Pediatric respiratory illnesses, including asthma, bronchiolitis, pneumonia, croup, and influenza, are among the most prevalent and costly conditions in US children’s hospitals, accounting for approximately one-third of pediatric hospitalizations and representing a leading cause of emergency department visits.^1–3^ Although clinicians can diagnose viral infections clinically, they frequently order viral testing to identify specific pathogens, and literature suggests this practice is increasing over time.^4,5^ This testing has a significant financial impact on healthcare, with one institution reporting that the comprehensive respiratory viral test accounted for an estimated two million dollars in charges from 2015 to 2017.^6^

Although some studies suggest that viral testing may assist in identifying clinical diagnoses and inform hospital infection-control practices, routine use raises concerns about overutilization and increased healthcare costs, with mixed evidence regarding its impact on patients’ clinical management.^4,5,7–14^ For example, up to 44% of children <1 year old and 27% of children <6 years old may test positive for a virus while only having asymptomatic colonization with postviral shedding.^15^ This observation may obscure the true etiology of their illness and lead to false reassurance with delayed identification of an underlying bacterial infection. Additionally, many children have co-infections that reduce the practical diagnostic value of viral testing, and clinicians often discontinue antibiotics based more on clinical factors than on viral testing alone.^9,11,14,16,17^ Studies have not demonstrated that viral testing has a significant impact on admission rates or inpatient length of stay.^14,18–20^ Additionally, viral testing increases antiviral prescribing rates, exposing children to potential side effects that can be worse than the initial presenting symptoms.^10,12,21^

In response to these concerns, the American Academy of Pediatrics Section on Emergency Medicine included the following recommendation in the 2022 Choosing Wisely Campaign: “Do not obtain comprehensive viral panel testing for patients who have suspected respiratory viral illness.” Clinicians should instead reserve testing for high-risk patients or situations in which results will guide treatment or public health decisions.^22^ Some examples of this would be at-risk children needing influenza treatment or in public health crises such as the COVID-19 pandemic. The Infectious Diseases Society of America and the American Society for Microbiology recommend narrow targeted viral testing for the general population instead of comprehensive viral testing, which should be reserved for the immunocompromised or severely ill.^23^

**Fig. 1.**
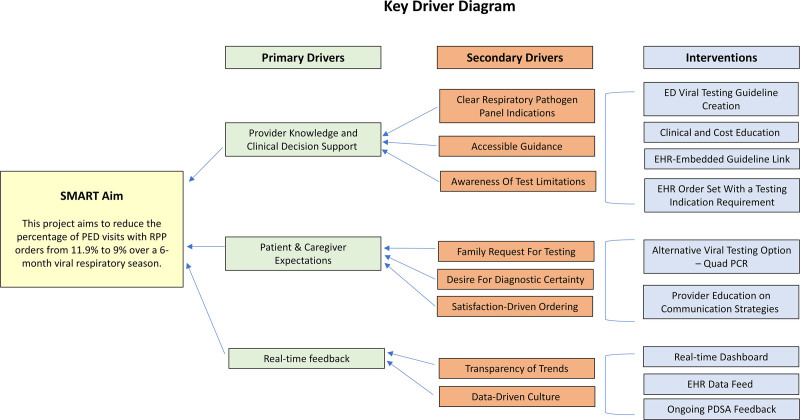
Key driver diagram. Key Driver Diagram investigating comprehensive RPP testing within the PED. It outlines factors that contribute to unnecessary RPP testing and the interventions used to address them.

Before this initiative, our pediatric emergency department (PED) used rapid influenza and COVID-19 PCR testing as alternative options to our 14-target panel viral respiratory pathogen PCR (RPP). Although it yielded results quickly, the influenza test had low sensitivity and frequently produced false negatives. The COVID-19 PCR was highly accurate but had a longer turnaround time. During the early planning phase of this project, we invited all clinicians in the Division of Emergency Medicine, comprising 63 physicians and 30 advanced practice practitioners, to participate in a roundtable session at the end of the standard monthly department meeting to discuss RPP testing practices. In addition to the commonly cited rationale in the literature, including support for medication discontinuation, antiviral prescribing, and reducing further medical interventions, participants noted that other emergency departments in our community offered respiratory syncytial virus (RSV) testing, which was only available at our institution as part of the RPP.^9,11,12,16–18,21^ Families requested RSV testing despite being counseled that the results were unlikely to change clinical management. Many PED team members reported obtaining RPPs to increase patient satisfaction. Using information from this roundtable, we identified key drivers and potential interventions (Fig. 1).

In alignment with high-value care principles, this quality improvement (QI) initiative aimed to reduce the overuse of RPP testing in our PED.^24^ Other pediatric improvement studies have shown success in reducing testing and improving care with standardized guidelines, order set creation, and provider education.^25,26^ Similarly, we wanted to shift the testing culture in our PED by creating a clinical guideline centered on proper viral testing, implementing a viral testing order set with a narrower-spectrum viral PCR panel that targets viruses of primary public health importance (influenza A/B, COVID-19, and RSV), introducing a required RPP testing indication checkbox, and enhancing education regarding the appropriate use of the RPP, cost education, and family communication techniques.

At our institution in the viral respiratory season (October to March) preceding project implementation, clinicians ordered RPPs in 11.9% of all PED visits, incurring $693,750 in testing costs for the hospital. A random review of 328 patient charts revealed that clinicians ordered 50% of these tests without a clear clinical indication. This project aimed to reduce the percentage of PED visits with RPP orders from 11.9% to 9% over a 6-month viral respiratory season. We chose this reduction because it was roughly half of the 50% of unnecessary tests while still acknowledging that many patients in the ED may continue to have a clinical indication for testing.

## METHODS

### Setting and Context

We conducted this QI initiative using the Institute for Healthcare Improvement’s Model for Improvement framework in the Division of Emergency Medicine at a large PED that sees more than 80,000 patients annually.^27^ The division includes pediatric emergency medicine subspecialists, pediatricians, fellows, residents, advanced practice providers, and medical students.

### Study Population

This project included all patients evaluated in our PED during the respiratory season (October to March) within our project timeframe of October 2022 to March 2025. During the 6-month baseline respiratory season before implementation (October 1, 2022, to March 31, 2023), the PED saw 46,692 patients. During the 6-month intervention phase (October 1, 2023, to March 31, 2024), the PED saw 43,283 patients, and during the postintervention phase (October 1, 2024, to March 31, 2025), the PED saw 40,241 patients.

### Interventions

In 2023, we formed a multidisciplinary team that included pediatric emergency medicine physicians, an infectious disease physician, a laboratory specialist, and pediatric residents. We identified key drivers and designed interventions using plan-do-study-act (PDSA) cycles (Fig. 1). These interventions included creating a clinical guideline for viral testing, implementing a narrower-spectrum Quad (Influenza A/B, COVID-19, and RSV) PCR test, performing provider education, and establishing a new viral testing order set with a required RPP testing indication that also served as an educational tool for providers.

We implemented our four major interventions over 2 PDSA cycles during the project timeframe. On October 19, 2023, we launched PDSA cycle 1 by publishing the PED viral testing guideline and initiating clinical and cost education, which we reinforced every few months throughout the intervention phase. On January 9, 2024, we launched PDSA cycle 2 by introducing the Quad PCR panel and adding an order set with a mandatory indication field requiring clinicians to document the indication for RPP testing. We implemented both PDSA cycle 2 interventions simultaneously due to IT timeline limitations.

### Clinical Guideline and Educational Outreach

**Fig. 2.**
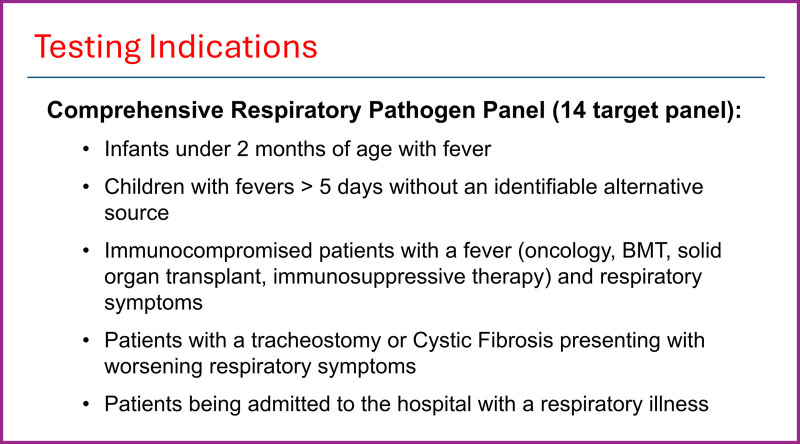
Testing indications for obtaining an RPP. Excerpt from the viral testing clinical guideline listing indications where it would be appropriate to order an RPP. Recommended testing should focus on patients at high risk or with test results that could change their clinical management. We posted the official document on our hospital intranet and embedded it in the viral testing order set for easy reference by providers.

We developed a clinical guideline to help clinicians determine when RPP testing is appropriate. Reflecting on the patient population at our institution, the guideline focused on high-risk patients or situations in which test results could change clinical management.^19,22,23^ Indications included infants younger than 2 months of age with fever; children with fever lasting more than 5 days without an identifiable source; immunocompromised patients with fever and respiratory symptoms; patients with tracheostomy or cystic fibrosis presenting with worsening respiratory symptoms; and any patient admitted to the hospital with a respiratory illness (Fig. 2). The clinical guideline was developed with the multidisciplinary team to ensure the project did not interfere with inpatient practices and improve buy-in.

Although routine viral respiratory testing is not indicated for all hospitalized patients with respiratory symptoms, certain inpatient services outside of the PED QI project preferred to continue testing. Given that these services care for higher-risk patient populations for whom viral identification may provide additional clinical value, we allowed them to continue testing without regulation and plan to reassess this approach in future projects. We designed the guideline to support, rather than replace, clinical judgment and allowed clinicians to order RPPs outside these indications when they deemed it appropriate.

Following institutional approval, we published the guideline on the hospital intranet and disseminated it through departmental and committee presentations and email communication. In addition to raising awareness of the guideline, we added cost information and family communication strategies. We directed educational outreach efforts to attending physicians, advanced practice providers, fellows, and residents, beginning with the rollout of PDSA cycle 1 and reinforced it every few months throughout the intervention phase.

### Narrow-spectrum Quad PCR

We partnered with the hospital laboratory to implement a narrow-spectrum Quad PCR that tests exclusively for influenza A/B, COVID-19, and RSV (Cepheid Xpert CoV-2/Flu/RSV). This test improved influenza testing sensitivity, provided a narrower-spectrum test option that allows clinicians to focus on viral infections most relevant to public health, and offered an alternative test that included RSV for families specifically requesting viral testing. The test has a shorter laboratory turnaround time than the comprehensive RPP (60 vs. 150 min) at a lower cost ($75/unit vs. $125/unit).

### PED Viral Testing Order Set with Required Testing Indication for the RPP order

We created a PED viral testing order set in the electronic health record (EHR) to centralize viral testing options (Sunrise Clinical Manager, Altera Digital Health, Chicago, Ill.). The order set was incorporated into all ED order sets that typically include this type of testing, with a link to the PED viral testing guideline for easy clinician access. Within the order set, the RPP test included a required testing indication section. This testing indication section must also be completed when ordering the RPP individually, outside any order set. If none of the recommended indications apply, clinicians may select “none of the above” to proceed with the order. We monitored EHR testing data via a clinical dashboard, and the QI team provided feedback to the PED team every couple of months via email and departmental meetings. The order set and RPP order also serve as a just-in-time education tool to guide appropriate RPP use, as the clinical indications field is required when placing the order. To support sustainability, clinicians have continued to have access to the clinical guideline and EHR order set beyond the QI project period.

### Study of the Intervention

We created a clinical dashboard to track intervention progress using EHR viral testing data from all PED visits. Our bioinformatics team built and validated the dashboard to ensure the accuracy and reliability of the reported data. This dashboard displays RPP testing relative to total PED visits and captures the indication selected by clinicians when ordering RPPs. It also tracks the use of the new Quad PCR and rapid influenza tests. Because COVID-19 PCR testing was already declining when this QI project began, as clinical practice shifted away from the pandemic era, we excluded this metric from the analysis to avoid data collection bias.

### Process Measure

To ensure our interventions targeted the intended patient population, we monitored the appropriateness of RPP orders. Before the rollout of the RPP testing indication order, the QI team conducted random chart reviews to assess clinical appropriateness in accordance with the viral testing guideline. If the patient did not meet any of the 5 reasons for testing, the test was deemed inappropriate.

Once the viral testing order set rolled out in PDSA 2, data on testing appropriateness were automatically collected from the required RPP testing indication field. All providers ordering RPP tests accessed the guideline-based clinical indications via the ED viral testing guideline link and completed the required indication selection process. A “none of the above” selection within the RPP order indicated that the patient did not meet approved testing criteria and likely represented an inappropriate test. We expected to see a reduction in these orders if the intervention effectively targeted the correct patient population.

For this metric, we measured the percentage of inappropriately ordered tests, defined as those with a “none of the above” selection or through chart review before order set implementation.

### Balancing Measures

To monitor unintended consequences of increased use of other viral tests while focusing on limiting RPP use, the team tracked ordering trends for other respiratory viral tests, specifically the rapid influenza and Quad PCR tests. These data showed the total percentage of ED visits, accounting for month-to-month variations in patient volume.

### Measures and Analysis

Because viral testing varies seasonally, with higher respiratory illness volumes during the winter respiratory season (October to March), we conducted year-to-year comparisons during this period to evaluate the intervention’s effectiveness. Data are presented as percentages of ED visits to adjust for variations in ED patient volumes. We obtained all data from the clinical dashboard.

Data were analyzed and graphed over time using statistical process control charts in Excel with the QI Charts plug-in (P’ capabilities). We shifted the centerline starting in October 2023, when values fell below the baseline lower control limits and all consecutive data points were below the baseline mean. RPP cost savings were calculated by multiplying the difference in absolute test counts by $125 per unit, the price the hospital was charged for the test at the time of our project.

### Ethical Considerations

A research determination committee classified this project as a QI project. The institutional review board therefore exempted the study from full review in accordance with institutional policy.

## RESULTS

During the 6-month baseline phase (October 1, 2022, to March 31, 2023), the PED saw 46,692 patients. During the QI intervention phase (October 1, 2023, to March 31, 2024), the PED evaluated 43,283 patients. In the postintervention phase (October 1, 2024, to March 31, 2025), the PED saw 40,241 patients.

### Primary Aim

**Fig. 3.**
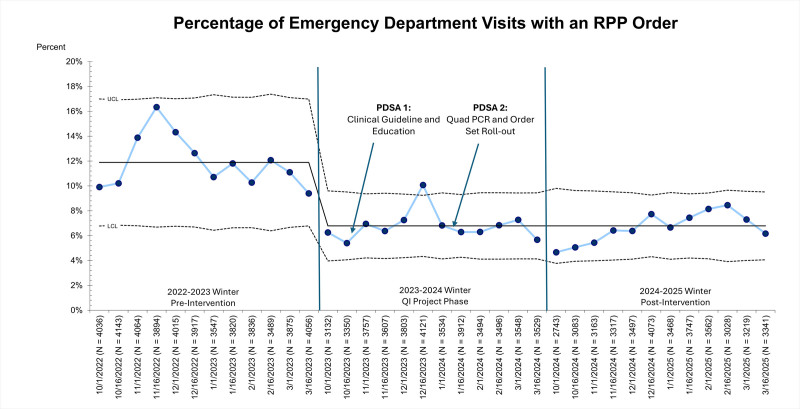
Percentage of emergency department visits with an RPP order. P-prime SPC showing the percentage of PED visits with an RPP order over time. PDSA cycle 1 was introduced on October 19, 2023, and PDSA cycle 2 was introduced on January 9, 2024. The 2 vertical lines separate the viral winter respiratory seasons, with the off-season months excluded from the data set. There was an improvement from 11.9% to 6.8% after the QI project implementation.

Across PDSA cycles 1 and 2, we reduced the percentage of PED visits with an RPP order from 11.9% to 6.8% (Fig. 3). This reduction was sustained in the subsequent winter season. This reduction resulted in $323,375 in hospital RPP testing cost savings during the QI intervention phase and $355,750 in savings the following year.

### Process Measure

The percentage of inappropriate RPP orders decreased from 50% at baseline to 28.5% after PDSA 1, then to 27% after PDSA 2. During the postintervention phase, the average percentage further decreased to 25%, demonstrating sustained improvement. Because the order set required clinicians to select an indication for testing, 100% of providers who ordered RPPs encountered the embedded educational component after PDSA 2 began.

### Balancing Measures

**Fig. 4.**
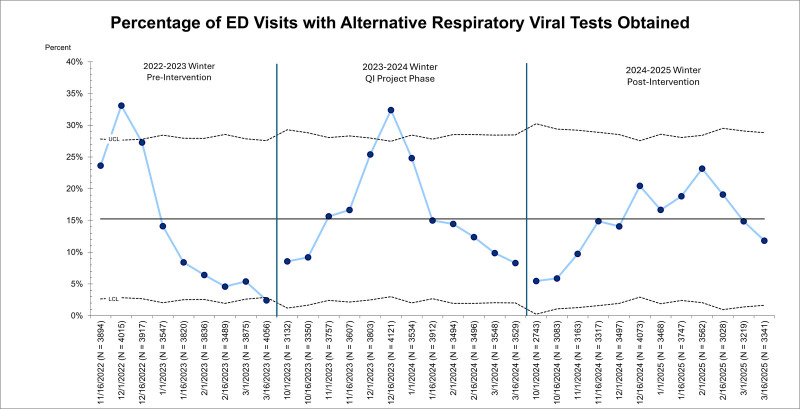
Percentage of PED visits with rapid influenza or Quad (RSV, COVID-19, influenza A/B) PCR testing. P-prime SPC chart showing the percentage of PED visits with alternative viral respiratory testing (rapid influenza and Quad PCR). The vertical lines denote the boundaries between winter respiratory seasons, excluding the off-season months. Time points from March 1, 2022, to November 16, 2022, were excluded due to testing supply chain disruptions at our facility. There were no significant increases in alternative viral testing associated with the QI efforts to reduce RPP use. Each year, testing peaked around the respiratory season, with a less pronounced peak in the postintervention phase (October 2024 to March 2025), suggesting a possible trend toward reduced alternative viral testing.

To monitor unintended increases in the use of other viral tests while limiting RPP use, the team tracked ordering trends for alternative respiratory viral tests, rapid influenza, and Quad PCR, throughout the intervention (Fig. 4). From March 1 to November 16, 2022, time points were excluded because no alternative testing options were available due to supply chain disruptions at our facility. After introducing the Quad PCR, the team observed an immediate and significant decrease in rapid influenza testing, and the Quad PCR became a consistent percentage of total viral testing in the PED. Notably, alternative viral testing did not increase as RPP use declined. Each year, testing peaked during the respiratory season, with a less significant peak in the postintervention phase, suggesting a possible trend toward reduced testing.

## DISCUSSION

This QI initiative significantly improved comprehensive viral PCR testing practices in the PED, achieving a sustained reduction in the percentage of PED visits with an RPP order from 11.9% to 6.8%. This reduction resulted in $323,375 in hospital RPP testing cost savings during the QI intervention season and $355,750 in savings the following year. By avoiding over 2,900 RPP nasopharyngeal swabs annually, this initiative likely reduced patient discomfort and stress.^28^

The RPP test has a longer laboratory turnaround time than alternative viral testing options (150 min RPP vs. 60 min Quad PCR vs. 15 min rapid influenza), which may impact ED length of stay. During this project, the average PED length of stay for all ED visits decreased from 350 minutes in the preintervention respiratory season to 298 minutes during the QI intervention season, with similar times demonstrated the following year. lthough reduced RPP use may have contributed to our shorter PED length of stay through overall RPP time savings, other throughput improvement measures were occurring during this time, and the overall decrease in patient volume prevents us from reliably presenting the improved length of stay as straightforward time savings.

Notably, the reduction in RPP orders was not associated with an increase in alternative viral testing. If anything, the postintervention phase demonstrated a lower peak in testing, suggesting a potential trend toward decreased use of alternative testing. This finding contrasts with a similar inpatient QI initiative project by Innis et al, in which reduced RPP testing led to increased narrow-spectrum testing.^19^ These results suggest an overall improvement in testing practices and a potential culture shift regarding the need for viral testing.

Consistent with prior QI initiatives, including those by Ostrow et al. and Innis et al., a multifaceted approach proved most effective in improving viral testing practices.^10,19^ We observed the greatest change in impact following the implementation of the clinical guideline and educational outreach. The most impactful component for sustaining improvement, however, was the implementation of the required testing indication field for RPP testing. This feature provides real-time decision support to clinical staff on appropriate indications for ordering the test and offers ongoing automated support for the initiative.

Although the initial implementation required substantial effort to develop the clinical guideline, data dashboard, and EHR order set, we sustained improvements with minimal ongoing resources. Maintenance primarily involved continued access to the automated EHR indication requirement and the clinical guideline. Our QI team has periodically reviewed dashboard data to monitor efforts and ensure accuracy, but the project has not required any additional intervention. In contrast, the QI initiative by Ostrow et al required ongoing, targeted decision support from QI team members for clinicians with outlier practices to ensure sustainability.^10^ Overall, our intervention had minimal impact on clinical workflow, requiring only a minor change with a few additional mouse clicks when ordering an RPP test.

### Limitations

This QI initiative was conducted at a single institution, which may limit generalizability. Parental requests for viral testing were a key driver of inappropriate RPP use. Although some interventions aimed to address this factor, including the Quad PCR and provider education on communication, the project did not include a formal assessment of patient or caregiver perceptions, nor did it evaluate potential effects on patient satisfaction.

Additionally, postintervention data on testing indications were based on clinician self-report and may be subject to reporting bias. To mitigate this limitation, the order set included a “none of the above” option, which accounted for approximately 25% of all orders. Because this option did not provide a clear advantage to clinicians, we believe reporting bias was minimal.

### Next Steps

Although this QI initiative focused on RPP ordering practices in the PED, data revealed that more than 30% of all RPPs were ordered for patients admitted with a respiratory illness, making inpatient care the most common setting for RPP testing. However, random chart reviews revealed that many of these tests were not clinically necessary and did not alter patient management. The next phase of this QI initiative will focus on reducing unnecessary RPP ordering practices in the inpatient hospital setting to improve diagnostic stewardship and enhance clinical efficiency across the continuum of care.

## CONCLUSIONS

This QI initiative significantly improved and sustained comprehensive viral PCR testing practices within the PED without increasing the use of alternative respiratory viral testing.

## ACKNOWLEDGMENTS

The authors thank Julie McGrath and Richard Engel, MD, for assisting with manuscript revisions and presentation of content.
